# Associations between sexual identity and caries risk indicators among adolescents and adults in Nigeria: implications for policy and actions

**DOI:** 10.3389/froh.2025.1551013

**Published:** 2025-04-29

**Authors:** Morenike Oluwatoyin Folayan, Olakunle Oginni, Olanrewaju Ibigbami, Abiola Adeniyi, Joanne Lusher

**Affiliations:** ^1^ Department of Child Dental Health, Obafemi Awolowo University, Ile-Ife, Nigeria; ^2^ Department of Mental Health, Obafemi Awolowo University, Ile-Ife, Nigeria; ^3^ Department of Child Dental Health, Lagos State University Teaching Hospital, Lagos, Nigeria; ^4^ Provost Office, Regent’s University London, London, United Kingdom

**Keywords:** sexual minority, heterosexual, tooth brushing, dental service utilisation, caries risk behaviour

## Abstract

**Background:**

There is little known about the oral health profile of sexual minority individuals in Africa. The study aimed to investigate the association between sexual identity and dental caries risk behaviors of adolescents and adults in Nigeria.

**Methods:**

This was a secondary analysis of data collected from participants aged 13 years and above recruited using an online electronic survey between 16th September and 31st October 2020. The dependent variable was dental caries risk behaviour (daily tooth brushing twice a day or more, daily consumption of refined carbohydrates in-between-meals thrice a day or more, history of dental service utilisation, and poor knowledge of dental caries prevention). The independent variable was sexual identity (heterosexual and sexual minority individuals). Four multivariate regression models were developed to assess the associations between sexual identity and each of the dependent variables. Each model was adjusted for age, sex, educational level, employment status, and marital status.

**Results:**

The data of 2,772 respondents were extracted for analysis. Sexual minority individuals had significantly lower odds of consuming refined carbohydrates in-between-meals three times daily or more (AOR: 0.53; 95% CI: 0.43–0.64; *p* < 0.001), tooth brushing twice daily or more (AOR: 0.82; 95% CI: 0.68–0.99; *p* = 0.037), and making use of a dental service within the last year before the survey (AOR: 0.66; 95% CI: 0.55–0.79; *p* < 0.001).

**Conclusion:**

While sexual minority individuals in Nigeria exhibit some protective behaviors (lower sugar intake), their reduced dental care utilization and brushing frequency signal a need for inclusive, equity-focused oral health policies. Further research is needed to provide evidence for the development of a comprehensive oral healthcare programme that addresses the needs of the sexual minority population.

## Introduction

Sexual minority individuals—people whose sexual orientation, gender identity, or expression differs from the dominant societal norms—face health disparities when compared to their heterosexual counterparts. Research consistently shows that they experience poorer overall health, regardless of age (1). Although studies on their oral health are limited, existing evidence suggests that while clinical assessments reveal no major differences by sexual orientation, sexual minority adults tend to rate their oral health more negatively than heterosexual adults (2). In addition, lower oral health literacy levels among sexual minorities are linked to reduced use of dental services (3).

Several risk factors contributing to poorer oral health in sexual minority individuals compared to their heterosexual counterparts have been identified. A key factor is the challenges sexual minority individuals often face in school settings, including feelings of unsafety, which can lead to lower attendance or higher dropout rates (4). Since education is a strong predictor of health, social, and behavioural outcomes (5), this disparity in educational attainment has long-term consequences. Specifically, lower education levels are linked to reduced oral health literacy (6), which is associated with worse oral health outcomes (7). However, the prevalence of oral diseases may differ across populations because of the influence of a complex interplay of biological, behavioural, socio-economic, and cultural factors (8, 9). These variations can be observed between countries, within regions of the same country, and even among different demographic groups [age affecting the biology of the teeth, behaviour, health and social risk factors (10–14), sex, employment status (15, 16), and marital status (17–20)].

In addition, sexual minority groups are at greater risk of contracting HIV than their heterosexual counterparts (21, 22). Sexual minority individuals often face significant barriers to accessing healthcare, including stigma and discrimination from healthcare providers, as well as a lack of provider awareness regarding their unique healthcare needs (23, 24). These barriers may also negatively impact their utilisation of dental services. Additionally, sexual minorities are at a higher risk for mental health challenges compared to heterosexual individuals (25), which can indirectly increase their risk of dental caries. Mental health issues may lead to poor oral hygiene and frequent consumption of refined carbohydrates between meals (26, 27), both of which are associated with a heightened risk of dental caries (28). Given the strong connection between oral health and overall health (29), it is crucial to implement targeted oral health interventions to enhance the well-being and quality of life of all individuals (30). Understanding the specific risk factors contributing to poor oral health in sexual minority populations is essential for designing effective preventive oral health strategies tailored to their needs.

One possible link between sexual minority status and dental caries risk is HIV infection. In Nigeria, the risk for HIV infection among sexual minority individuals is very high (31). HIV infection is associated with poorer oral health outcomes (32, 33), and individuals living with HIV are more susceptible to dental caries compared to the general population (34, 35). However, in Nigeria, studies examining the link between HIV status and dental caries have focused on children (36, 37), with limited research on adolescents, adults, and other HIV-affected sub-populations.

A few countries have healthcare interventions and programmes for sexual minority individuals. However, in Nigeria, there are no national programmes specifically designed for this population. The socio-political landscape is marked by stringent laws against sexual minority individuals that fuel pervasive cultural and religion-informed stigma and discrimination against the community (38). These factors exacerbate health disparities, including limited access to healthcare and higher HIV prevalence among sexual minority populations (39). Instead, the health-related needs of sexual minority individuals are primarily addressed by non-governmental organizations.

In a context where oral healthcare resources are limited, prioritizing interventions becomes especially critical. Given the potential link between sexual identity and poor oral health, it is essential to explore the association between sexual identity and dental caries risk among sexual minority adolescents and adults in Nigeria. Identifying groups at higher risk for poor oral health can guide the allocation of scarce oral health resources effectively. This study aimed to examine whether an association exists between dental caries risk behaviour and sexual orientation among adolescents and adults in Nigeria. The hypothesis posited that sexual minority individuals are more likely to engage in behaviours that promote dental caries development compared to their heterosexual counterparts.

## Methods

### Study design

The current study was a secondary analysis of primary cross-sectional data collected on the general, oral, mental, and sexual health of adolescents and adults in Nigeria. Details of the primary study have been published previously (40).

### Study setting

This study was conducted in Nigeria, and data was collected from the 36 states of the country.

### Study population

Participants were recruited nationwide for a cross-sectional survey conducted via an online electronic questionnaire (SurveyMonkey^©^). Individuals aged 13 years and above who provided informed consent—or assent, where applicable—were eligible to participate. There were no exclusion criteria.

### Sample size

Data from 2,772 respondents (78.5% of the 3,529 who accessed the questionnaire) were successfully extracted. The sample size was deemed statistically adequate as each dependent variable in the study had responses from at least 10 participants. This allowed for regression analyses to be conducted with a minimum significance level of 0.05 (41, 42).

### Recruitment of study participants

The study employed multiple non-probability sampling techniques, including exponential non-discriminatory snowball sampling (43) and crowdsourcing. Twenty-nine individuals working with sexual minority communities reached out to both heterosexual and sexual minority individuals, leveraging social media platforms to establish contacts. The survey link was distributed via Facebook, Twitter, Instagram, social network email lists, and WhatsApp groups. In addition, the lead non-governmental organization, Total Health Empowerment and Development Initiative, collaborated with partners to disseminate the survey links through their networks.

The survey began with a brief introduction outlining the voluntary nature of participation, the study's purpose, and the confidentiality of the data. Administered in English, the questionnaire took approximately 15 min to complete, with each participant allowed to submit only one response.

### Data collection process

Data collection occurred between September 16 and October 31, 2020. Data were collected via an online electronic survey (SurveyMonkey) conducted between 16 September and 31 October 2020. Participants aged 13 years and above were eligible to participate, with data from respondents aged 13–19 years extracted for this analysis. A respondent-driven sampling strategy was employed, beginning with 23 peer educators and 6 study staff who disseminated the survey link within their networks. Recruitment was further supported through convenience sampling using social media platforms, email lists, and WhatsApp groups. To enhance accessibility, open links were also made available at NGO offices where peer educators assisted individuals with low literacy in completing the survey. The self-administered questionnaire was in English and took an average of 15 min to complete. Participation was voluntary and anonymous, and informed consent was obtained.

### Data variables

For this study, the questionnaire developed by Khami et al. (44) was adapted to evaluate preventive oral practices. This same questionnaire has been used in several studies conducted in Nigeria (45, 46). It was self-administered and included fixed-response options.

### Dependent variables

#### Tooth-brushing

Respondents were asked to indicate how often they brush their teeth daily, with the following options: “Irregularly or never,” “Once a week,” “A few (2–3) times a week,” “Once a day,” and “More than once a day.” For the binary regression analysis, “More than once a day” was used as the reference category, by the World Health Organization's guidelines on recommended tooth brushing frequency (47). Respondents were then categorized as “Yes” (brushing more than once a day) or “No” [brushing irregularly or never, once a week, a few (2–3) times a week, or once a day].

#### Consumption of refined carbohydrates in-between-meals

Respondents were asked to report the frequency of consuming sugar-containing snacks or drinks between main meals, with the following response options: “About 3 times a day or more,” “About twice a day,” “About once a day,” “Occasionally,” “Not every day,” and “Rarely or never eat between meals.” For the binary regression analysis, the category “About 3 times a day or more” was selected as the reference value, as it represents the threshold for dental caries development in children in Nigeria (48). Based on this, participants were classified into two groups: “Yes” (consuming refined carbohydrates between meals about 3 times a day or more) and “No” (consuming refined carbohydrates about twice a day, about once a day, occasionally, not every day, rarely, or never).

#### Dental service utilisation

Respondents were asked to specify the timing of their most recent dental check-up, choosing from the following options: “Within the last 6 months,” “More than 6 months but up to one year ago,” “More than 1–2 years ago,” “More than 2–5 years ago,” “More than 5 years ago,” “Never,” or “Do not remember.” Attending a dental check-up within the past year was considered as receiving preventive care. In the binary regression analysis, the reference category was a dental check-up within 6–12 months (48). Participants were then categorized into two groups: “yes” (those who had a check-up within the last 6 months or more than 6 months but less than one year ago) and “no” (those who had a check-up more than 1–2 years ago, more than 2–5 years ago, more than 5 years ago, never, or could not remember).

#### Knowledge of dental caries prevention

This variable was assessed using the methodology outlined by Folayan et al. (45). Respondents were asked to respond to eight statements using a five-point Likert scale, ranging from “strongly agree” to “agree,” “disagree,” “strongly disagree,” and “do not know.” Responses were assigned scores from 1 to 5, with “strongly agree” given a score of 5 and “do not know” a score of 1. In cases where no response was provided, a score of 1 was allocated. The total score could range from 8 to 40. The mean of the final scores was used as the cut-off point, with respondents scoring below the median classified as having poor knowledge, while those with scores equal to or above the median were categorized as having good knowledge. The mean score for this sample was 23.4.

### Independent variable

#### Sexual identity

Respondents were asked to identify their sexual orientation as heterosexual/straight, gay, lesbian, bisexual, or prefer not to say. The term “sexual minority individuals” is used as an umbrella to refer to those who are attracted to the same sex or to both sexes. Consequently, individuals who identified as gay, lesbian, or bisexual were categorized as “sexual minority”.

### Confounders

#### Sociodemographic and other relevant variables

Age at last birthday (in years), sex at birth (male, female, intersex, decline to answer), education level completed (no formal education, primary, secondary, tertiary), employment status (employed, not employed) and marital status (single, married/co-habiting and separated/divorced). We categorised sex at birth into male and non-male (female, intersex, decline to answer) for the regression analysis. We also obtained information on the participants’ HIV status (positive, negative, I do not know).

### Data analysis

Analyses were carried out using SPSS (version 26). Descriptive statistics were computed for all the study variables as means and standard deviations for numerical variables or as frequencies and percentages for categorical variables. We specified multivariate logistic regression models to determine the associations between sexual identity (heterosexual, sexual minority) and the explanatory variables. The models were adjusted for the listed sociodemographic variables.

### Ethical considerations

Ethical approval was obtained from the Health Research Ethics Committee of the Institute of Public Health at Obafemi Awolowo University in Ile-Ife, Nigeria (IPHOAU/12/1571). Parental waiver for consent for adolescents 13–17 years old was obtained in Nigeria in line with the national guidelines on sexual and reproductive health research conduct with adolescents (49). All study participants who took the online survey were offered compensation for internet data of N100 (USD 0.27).

## Results

The age of the study participants ranged from 13 to 62 years, with a mean (standard deviation) of 20.90 (5.55) years. Among the respondents, 1,501 (54.1%) identified as heterosexual, 170 (6.1%) identified as HIV-positive, and 1,290 (46.5%) had completed secondary school education. Additionally, 2,358 (85.1%) were single, 421 (15.2%) were unemployed, and 1,587 (57.3%) were students. Furthermore, 631 (22.8%) reported consuming refined carbohydrates between meals three times daily or more, 803 (29.0%) brushed their teeth twice daily or more, 852 (30.7%) had used dental services within the last year, and 1,418 (51.2%) had good knowledge of dental caries prevention.

As shown in Table 1, a significantly larger proportion of respondents identifying as sexual minorities reported being unaware of their HIV status (*p* < 0.001), being HIV-positive (*p* < 0.001), lacking formal education (*p* < 0.001), and being unemployed (*p* < 0.001). In addition, a significantly lower proportion of sexual minority individuals had a tertiary education (*p* < 0.001).

**Table 1:** Association between sexual identity and the independent, dependent, and confounding variables (*N* = 2,772).

Variables	Heterosexuals	Sexual minority individuals	*p*-value
	*N* = 1,501	*N* = 1,271	
Age in years	20.8 (4.9)	20.8 (5.9)	0.182
HIV status			
Negative	1,144 (76.2)	746 (58.7)	<0.001
Unknown	320 (21.3)	392 (30.8)	
Positive	37 (2.5)	133 (10.5)	
Educational status			
No formal education	39 (2.6)	90 (7.1)	<0.001
Primary education	52 (3.5)	33 (2.6)	
Secondary education	624 (41.6)	666 (52.4)	
Tertiary education	786 (52.4)	482 (37.9)	
Employment status			
Employed	349 (23.3)	394 (31.0)	<0.001
Student	962 (64.1)	625 (49.2)	
Retired	15 (1.0)	6 (0.5)	
Unemployed	175 (11.7)	246 (19.4)	
Marital status			
Single	1,297 (86.4)	1,061 (83.5)	0.065
Married/cohabiting	180 (12.0)	179 (14.1)	
Separated/divorced	24 (1.6)	31 (2.4)	

Table 2 shows that sexual minority individuals had significantly lower odds of consuming refined carbohydrates in-between-meals three times daily or more (AOR: 0.53; 95% CI: 0.43–0.64 *p* < 0.001), tooth brushing twice daily or more (AOR: 0.82; 95% CI: 0.68–0.99; *p* = 0.037), and making use of a dental service within the last year before the survey (AOR: 0.66; 95% CI: 0.55–0.79; *p* < 0.001). Although they also had lower odds of having good knowledge of dental caries prevention, this association was not found to be significant (AOR: 0.87; 95% CI: 0.74–1.02; *p* = 0.095).

**Table 2:** Binary logistic regression analysis to determine factors associated with the oral health status of sexual minority individuals and heterosexuals in Nigeria (*N* = 2,772).

Variables	Total n (%)	Sugar consumption between meals three times a day or more			Tooth brushing twice a day or more			Dental service utilisation in the last 12 months			Knowledge of dental caries prevention		
		Yes *n* = 631	No *n* = 2,141	AOR (95% CI) *p*-value	Yes *n* = 803	No *n* = 1,969	AOR (95% CI) *p*-value	Yes *n* = 852	No *n* = 1,920	AOR (95% CI) *p*-value	Poor *n* = 1,354	Good *n* = 1,418	AOR (95% CI) *p*-value
Sexual identity													
Heterosexual	1,501 (54.1)	426 (67.5)	1,075 (50.2)	1.00	471 (58.7)	1,030 (52.3)	1.00	544 (63.8)	957 (49.8)	1.00	651 (50.8)	805 (56.8)	1.00
Sexual minority	1,271 (45.9)	205 (32.5)	1,066 (49.8)	0.53 (0.43–0.64) <0.001	332 (41.3)	939 (47.7)	0.82 (0.68–0.99) 0.037	308 (36.2)	963 (52.2)	0.66 (0.55–0.79) <0.001	631 (49.2)	613 (43.2)	0.87 (0.74–1.02) 0.095
HIV status													
HIV negative	1,890 (68.2)	426 (67.5)	1,464 (68.4)	1.00	645 (80.3)	1,245 (63.2)	1.00	671 (78.8)	1,219 (63.5)	1.00	815 (63.6)	1,033 (72.8)	1.00
HIV Unknown	712 (25.7)	168 (26.6)	544 (25.4)	1.45 (0.97–2.18) 0.070	100 (12.5)	612 (3.1)	0.43 (0.34–0.56) <0.001	122 (14.3)	590 (30.7)	0.35 (0.27–0.44) <0.001	390 (30.4)	297 (20.9)	0.69 (0.57–0.84) <0.001
HIV positive	170 (6.1)	37 (5.9)	133 (6.2)	1.06 (0.84–1.33) 0.642	58 (7.2)	112 (5.7)	1.04 (0.73–1.48) 0.852	59 (6.9)	111 (5.8)	1.34 (0.94–1.89) 0.102	77 (6.0)	88 (6.2)	0.98 (0.70–1.36) 0.894
Age in years (mean (SD)	20.90 (5.55)	19.35 (4.28)	21.36 (5.80)	0.92 (0.89–0.95) <0.001	22.60 (5.99)	20.21 (5.2)	1.0 (0.98–1.02) 0.77	20.09 (4.66)	21.26 (5.87)	0.95 (0.93–0.97) <0.001	20.37 (5.38)	21.39 (5.66)	1.02 (1.00–1.04) 0.041
Educational status													
No formal education	129 (4.7)	19 (3.0)	110 (5.1)	1.00	13 (1.6)	116 (5.9)	1.00	34 (4.0)	95 (4.9)	1.00	57 (4.4)	69 (4.9)	1.00
Primary education	85 (3.1)	19 (3.0)	66 (3.1)	1.10 (0.52–2.29) 0.808	16 (2.0)	69 (3.5)	2.27 (1.00–5.16) 0.05	29 (3.4)	56 (2.9)	0.75 (0.39–1.41) 0.365	42 (3.3)	38 (2.7)	0.61 (0.34–1.09) 0.097
Secondary education	1,290 (46.5)	307 (48.7)	983 (45.9)	1.47 (0.86–2.54) 0.161	258 (32.1)	1,032 (52.4)	2.37 (1.27–4.41) 0.006	363 (42.6)	927 (48.3)	0.54 (0.34–0.85) 0.008	693 (54.1)	565 (39.8)	0.51 (0.34–0.77) 0.001
Tertiary education	1,268 (45.7)	286 (45.3)	982 (45.9)	2.17 (1.23–3.83) 0.007	516 (64.3)	752 (38.2)	4.35 (2.34–8.10) <0.001	426 (50.0)	842 (43.9)	0.80 (0.50–1.29) 0.361	490 (38.2)	746 (52.6)	0.83 (0.55–1.25) 0.364
Employment status													
Employed	743 (26.8)	117 (18.5)	626 (29.2)	1.00	305 (38.0)	438 (22.2)	1.00	177 (20.8)	566 (29.5)	1.00	341 (26.6)	381 (26.9)	1.00
Student	1,587 (57.3)	431 (68.3)	1,156 (72.8)	1.05 (0.80–1.38) 0.721	384 (47.8)	1,203 (61.1)	0.66 (0.52–0.84) 0.001	573 (67.3)	1,014 (52.8)	1.48 (1.16–1.89) 0.002	727 (56.7)	820 (57.8)	1.48 (1.19–1.85) <0.001
Retired	21 (0.8)	2 (0.3)	19 (0.9)	0.35 (0.08–1.57) 0.169	6 (0.7)	15 (0.8)	0.54 (0.20–1.49) 0.232	3 (0.4)	18 (0.9)	0.40 (0.11–1.43) 0.160	11 (0.9)	10 (0.7)	0.85 (0.35–2.09) 0.725
Unemployed	421 (15.2)	81 (12.8)	340 (15.9)	1.04 (0.75–1.46) 0.806	108 (13.4)	313 (15.9)	0.92 (0.68–1.22) 0.548	99 (11.6)	322 (16.8)	1.04 (0.77–1.41) 0.799	203 (15.8)	207 (14.6)	1.21 (0.93–1.58) 0.153
Marital status													
Single	2,358 (85.1)	597 (94.6)	1,761 (82.3)	1.00	576 (71.7)	1,782 (90.5)	1.00	766 (89.9)	1,592 (82.9)	1.00	1,125 (87.8)	1,170 (82.5)	1.00
Married/cohabiting	359 (13.0)	26 (4.1)	333 (15.6)	0.31 (0.20–0.47) <0.001	208 (25.9)	151 (7.7)	3.37 (2.62–4.34) <0.001	70 (8.2)	289 (15.1)	0.59 (0.44–0.80) 0.001	130 (10.1)	220 (15.5)	1.43 (1.11–1.83) 0.005
Separated/divorced	55 (2.0)	8 (1.3)	47 (2.2)	0.71 (0.33–1.55) 0.392	19 (2.4)	36 (1.8)	1.27 (0.71–2.29) 0.419	16 (1.9)	39 (2.0)	1.04 (0.56–1.92) 0.901	27 (2.1)	28 (2.0)	0.87 (0.50–1.51) 0.621
Nagelkerke R2	–	–	0.101	–	–	0.166	–	–	0.112	–	–	0.050	–
Omnibus test of model coefficients	–	–	190.52	<0.001	–	343.20	<0.001	–	229.71	<0.001	–	102.67	<0.001

## Discussion

This current study is the first to determine the oral health profile of sexual minority individuals in Nigeria and Sub-Saharan Africa. The findings indicate disparities in dental caries risk behaviors between heterosexual and sexual minority individuals. Sexual minority individuals have lower odds of brushing their teeth twice daily or more, visiting a dental service within the year before the survey, and consuming refined carbohydrates between meals three times a day or more. There was no significant difference in dental caries prevention knowledge based on sexual identity. These results provide partial support for the study hypothesis.

The main strengths of this study include the large sample size and the large representation of sexual minority individuals. However, the use of convenience sampling for participant recruitment limits the generalizability of the findings. Moreover, online recruitment may have led to a higher participation rate among individuals with secondary and tertiary education, as the survey tool likely reached those with smartphones and internet access. While mobile phone penetration in Nigeria is high (50), the use of smartphones is more prevalent among those with higher education, as they are more likely to have the income to afford and maintain smartphones and internet access. We were, however, constrained to this recruitment method due to the COVID-19 pandemic, which prevented face-to-face participant recruitment. On the positive side, the online approach allowed for broader national coverage, reaching participants from most states in Nigeria. The cross-sectional nature of the study also limits our ability to establish causal relationships. In addition, the self-reported data on dental caries prevention behaviours may not accurately reflect clinically assessed risk, as self-reporting is susceptible to social desirability bias.

While this study does not provide conclusive evidence that dental caries risk factors differ significantly between sexual minority and heterosexual individuals due to the limitations identified, the study, however, offers insights for generating hypotheses that can be further tested. This is particularly important, considering the complex relationships between dental caries risk factors. Poor oral hygiene contributes to dental caries development, especially when refined carbohydrate consumption is high (51). Regular dental visits play a crucial role in early dental caries detection and offer healthcare providers the opportunity to reinforce positive oral health behaviours. Our study finding suggests that, although sexual minority individuals report brushing their teeth less frequently than their heterosexual counterparts, their lower consumption of refined carbohydrates in between meals may reduce their dental caries risk. Conversely, the lower utilisation of dental services among sexual minority individuals could increase dental caries risk, as it limits the early diagnosis and timely treatment of dental caries, alongside reduced access to oral health information.

Although dental service utilisation is generally low among the Nigerian population, as hospital visits are often focused on curative rather than palliative care (52), the even lower utilisation among sexual minority individuals compared to heterosexuals may stem from safety concerns in public spaces. In Nigeria, the law imposes life imprisonment on individuals who engage in same-sex practices (53), fostering a culture of silence around sexual identity. In addition, public hospital staff are often perceived as judgmental and likely to discriminate against sexual minority individuals (54, 55). These factors discourage sexual minority individuals from seeking healthcare services (56), with severe implications for their health and well-being. The lower healthcare utilisation by sexual minority individuals highlights the need for oral health promotion initiatives tailored to their needs. Given that sexual minority individuals are not legally recognized in Nigeria and may not receive focused attention in the national public health programmes, integrating oral health education into existing health education programmes for sexual minority individuals may be the appropriate implementation strategy. Studies are needed on how to design and implement oral health programmes for sexual minority individuals in Nigeria.

The consumption of refined carbohydrates in between meals less than three times a day is a positive finding. High sugar consumption contributes to mental health challenges, including the increased risk for depression and suicidal ideation (57). Sexual minority individuals have a high risk for poor mental health (40). The current study finding suggests that the lower frequency of sugar consumption may not only be protective for caries prevention in the population but may also be linked to improved mental health outcomes. We hypothesize that limiting in-between-meal sugar intake to fewer than three times daily may protect against mental health challenges in sexual minority individuals while also reducing caries risk. This needs further exploration as these synergistic findings on oral health and mental health for sexual minority individuals may strengthen health education messages for the population. Further studies are needed to explore this hypothesis.

This study highlights the need for targeted actions to address the oral health care needs of sexual minority individuals in Nigeria and possibly other countries in Sub-Saharan Africa with similar political and social profiles like Nigeria. Key implications include advocating for inclusive health policies to eliminate legal and social barriers for access of sexual minority individuals to oral health care, integrating oral health education into existing health programmes that target sexual minority individuals like HIV prevention programmes, and improving community engagement programmes on oral health through the training of community healthcare provider training on oral health care education competence. Innovative service delivery models, such as mobile clinics and telehealth, are also essential for improving oral health access by hard-to-reach populations like sexual minority individuals. Public health campaigns should address dental caries risk factors while promoting oral hygiene and regular check-ups. Further research using robust sampling methods is necessary to validate the current findings, and policy advocacy may need to focus on legal reforms to address the criminalization of sexual minority identities, as the discriminatory laws may be contributing to poor dental service utilisation.

## Conclusion

While sexual minority individuals in Nigeria exhibit some protective behaviours (e.g., lower sugar intake), their reduced dental care utilisation and brushing frequency signal a need for inclusive, equity-focused oral health policies. Addressing these disparities requires multisectoral collaboration between public health agencies, sexual minority individuals’ advocacy groups, and dental care providers to ensure equitable access to care. Further research is needed to provide evidence for the development of a comprehensive oral healthcare programme that addresses the needs of the sexual minority population.

## Data Availability

The raw data supporting the conclusions of this article will be made available by the authors, without undue reservation.
